# A glimpse of the ERM proteins

**DOI:** 10.1186/s12929-016-0246-3

**Published:** 2016-03-17

**Authors:** Godwin A. Ponuwei

**Affiliations:** Cell migration laboratory, Molecular and Cellular Medicine Unit, Department of Biomedical Sciences, School of Biological Sciences, Hopkins Building, University of Reading, Whiteknights, Berkshire UK; Molecular and Cellular Medicine unit, Department of Biomedical sciences, School of Life Sciences, Hopkins Building, Whiteknights Campus, University of Reading, Reading, Berkshire UK

**Keywords:** Ezrin, Radixin, Moesin, Plasma membrane, Phospholipids, Cancer

## Abstract

In all eukaryotes, the plasma membrane is critically important as it maintains the architectural integrity of the cell. Proper anchorage and interaction between the plasma membrane and the cytoskeleton is critical for normal cellular processes. The ERM (ezrin-radixin-moesin) proteins are a class of highly homologous proteins involved in linking the plasma membrane to the cortical actin cytoskeleton. This review takes a succinct look at the biology of the ERM proteins including their structure and function. Current reports on their regulation that leads to activation and deactivation was examined before taking a look at the different interacting partners. Finally, emerging roles of each of the ERM family members in cancer was highlighted.

## Review

The ERM proteins are evolutionary conserved group of three related proteins (ezrin, radixin and moesin) that possess band Four point one (4.1) as a common origin [1]. They interact with the plasma membrane through a common FERM (Four point one, ERM) domain [2]. The ERM proteins are located in cellular structures such as filopodia, lamellipodia, apical microvilli, ruffling membranes, cleavage furrow of mitotic cells, retraction fibres, and adhesion sites, where the plasma membrane interacts with F-actin [3]. ERMs are critical for structural stability and for maintaining the integrity of the cell cortex by coupling transmembrane proteins to the actin cytoskeleton [1]. These proteins also play very pivotal intracellular scaffolding roles that aid signal transduction between the intracellular and extracellular compartments of the cell as well as interacting with other membrane phospholipids [4]. Thus, ERMs are involved in regulating several cellular processes including reorganization of actin cytoskeleton, cell survival, membrane dynamics, cell migration, adhesion and regulation of membrane protrusion [4, 5].

## Background

### Structure of ERM proteins

**Fig. 1:**
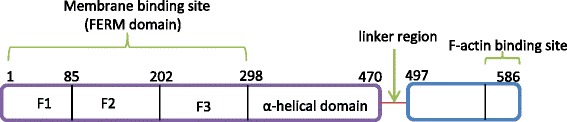
Basic Structure of ERM Proteins. All ERM proteins possess similar domain structure with the N terminus having F1, F2 and F3 subdomains. At the central portion of the protein is an α-helical domain which is followed by a linker region. At the last 30 carboxyl terminal end is the F-actin binding site

Structurally, at the amino terminus of ERM proteins is an approximately 1-296 amino acid FERM domain also known as N-terminal ERM association domain (N-ERMAD) through which they interact with cell membranes. X-ray crystallography revealed that the FERM domain consists of F1, F2 and F3, also respectively referred to as A, B and C subdomains that fold and joined together to form an cloverleaf structure, and these subdomains are homologous to ubiquitin, acyl-CoA binding protein and plekstrin homology domains respectively [4, 5] (Fig. 1). The FERM region is closely flanked by a central (approximately 200 amino acid) α–helical domain that form coiled coils [4] and mediate interaction with protein kinase A (PKA) [6]. The carboxylic terminal tail consists of 107 residues, and this terminus contains the F-actin binding site through which ERMs interact with the actin cytoskeleton [7]. In all ERM family members, distinct domains within the N-terminal head and C-terminal tail known as N- and C-ezrin-radixin-moesin association domains (N-ERMAD and C-ERMAD respectively) mediates homotypic and heterotypic head-to-tail interaction [4, 8, 9]. The N-ERMAD is distinct from the C-ERMAD in that it is a labile domain that is inactivated by chemical agents such as sodium dodecyl sulfate (SDS) treatment, and its activity is negatively affected by freeze thawing, whereas the C-ERMAD is unaffected by chemical treatment [10].

ERMs exist in a dormant, inactive closed conformation within the cytosol in which the C-ERMAD stretches from the F-actin binding site through F2 and F3 to part of the FERM region, thereby concealing both the F-actin and the membrane binding sites from other binding partners [7, 10, 11]. This covering of FERM by the C-ERMAD is bolstered by the central α-helical domain in that it binds the FERM domain to facilitate masking of both domains [4]. Activation of ERMs requires opening up the binding sites in the FERM domain and those of the F-actin biding sites in the C-terminal domain. This is achieved by phosphatidylinositol 4,5-bisphosphate (PIP_2_)-mediated uncoupling of the C-terminal domain from the FERM domain [1].

### Regulation of ERM proteins

ERM proteins function is regulated by a two-step process of open (active) and closed (inactive) conformation [12]. They are mainly regulated through conformational changes induced by phospholipids and kinases-mediated phosphorylation, and this results in activation of the proteins [13]. Recruitment of ERMs to areas of the plasma membrane containing high amount of phosphoinositides such as PIP_2_ exposes a conserved regulatory threonine phosphorylation residue (T567, T564 and T558 in ezrin, radixin and moesin respectively) located in the C-ERMAD domain [14], and this induces a successive activation mechanism whereby PIP_2_ first bind to a subdomain in the N-terminal FERM domain followed by plasma membrane translocation and phosphorylation of the threonine residues [15]. There are three lysine-rich consensus sites known to bind phosphoinositides on the FERM domain of ERM proteins and mutation on any of these sites inhibited PIP_2_-ERM interaction and translocation to the plasma membrane [16]. PIP_2_-mediated recruitment of ERMs to the plasma membrane is sufficient not only in the phosphorylation of these proteins by other kinases, but also in the formation of microvilli [17].

Phosphorylation of the conserved threonine residue can be induced by different signaling protein kinases such as Rho-associated protein kinase (ROCK), myotonic dystrophy kinase-related Cdc42-binding kinase, G-protein coupled receptor kinase 2, Nck interacting kinase [14, 18–20], protein kinase C (PKCα, PKCβ), NFкB-inducing kinase (NIK), lymphocyte-oriented kinase (LOK) thereby creating stearic hindrance that keeps the FERM and C-ERMAD domains apart [11, 21–23], and this stabilizes the active state of ERM proteins in their open conformation [15, 17]. Ezrin can be phosphorylated by cyclic-dependent kinase 5 (CDK5) on threonine 235 which lies between the FERM and C-ERMAD domains, and mutation of this site facilitated ezrin localization to plasma membrane [24].

Binding of ERM proteins to the cytoskeleton in many cases is strengthened by phosphorylation of the proteins [25]. Activation of the small Rho GTPase, RhoA and not Rac or Cdc42 was able to induce phosphorylation of both radixin and moesin, and this paralleled formation of membrane protrusions in Swiss 3 T3 cells [26]. Also upon activation of platelets with thrombin, the phosphorylation status of moesin on threonine 558 (a residue also phosphorylated by PKCθ) was enhanced and this bolstered the interaction of moesin with the cytoskeleton, and moesin was found localized at the spreading filopodia [27]. Phosphorylation of radixin on threonine 564 at the C-terminal half by Rho-kinase had no effect on the C-ERMAD to bind F-actin, but attenuated the ability of the C-ERMAD to bind N-ERMAD [28] suggesting that the activated state of ERM proteins during which the intramolecular interaction between the N- and C- terminal domains is inhibited, can be sustained by the phosphorylation of threonine 564 in radixin [25], threonines 558 and 567 in moesin and ezrin respectively [29].

ERM proteins can also be phosphorylated by receptor tyrosine kinases. Epidermal growth factor (EGF) receptor can phosphorylate ezrin at tyrosines 145 and 353 (Y145 and Y353) [30]. In epithelial kidney cells, Y353 phosphorylation is required for not only binding of phosphatidylinositol 3-kinase to ezrin, but for the activation of Akt signaling pathway [31]. Similarly, stimulation of ezrin-transfected LLC-PK1 cells with hepatocyte growth factor (HGF) resulted in increased phosphorylation of ezrin at the same tyrosine residues and this not only promoted cell migration, but also enhanced intracellular signal transduction [32]. Ezrin Y145 phosphorylation was demonstrated in Jurkat T-cells expressing Lck (a Src family kinase), but not in Lck-deficient cells [33].

It is now known that ERM proteins are also activated by sphingolipids. For instance in several cell lines such as A549, HEK, MEF, MCF7 and MDA cells, expression of the bioactive sphingolipid, sphingosine-1-phosphate (S1P) both endogenously and exogenously resulted in phosphorylation and activation of ezrin in a time- and dose-dependent manner [34, 35], and in Hela cells, S1P-mediated phosphorylation was found to be through S1P receptor 2 (S1PR2). This was required for filopodia formation [34]. In a PKC-dependent manner, S1P stimulation of pulmonary endothelial cells resulted in activation of ezrin and moesin, but not radixin [36, 37]. However, contrary to its known functions, via unclear mechanisms, S1P phosphorylation of ezrin resulted in inhibition of cell invasion, and this could be attributed to the ability of S1P to act on different receptors [35]. In Hela cells, generation of plasma membrane ceramide through breakdown of sphingomyelin by the action of sphingomyelinase resulted in dephosphorylation of ERM proteins, while decreasing plasma membrane levels of the sphingolipid resulted in ERM proteins hyperphosphorylation [12].

Regulation of ERM proteins is also brought about by dephosphorylation and inactivation of the proteins through the activities of phosphatases, and via PIP_2_ hydrolysis. In Hela cells, ceramide drives ERM dephosphorylation through activation of protein phosphatase 1α (PP1α) with the resultant effect of inactivating ERM and subsequent dissociation from the plasma membrane [38]. Similarly, overexpression of the small protein tyrosine phosphatase, phosphatase of regenerating liver-3 (PRL-3) in HCT116 colon cancer cell line resulted in dephosphorylation of ezrin [39]. Moesin can be downregulated by myosin light chain phosphatase through dephosphorylation of threonine 558 [40]. Although, in phorbol 12-myristate 13 acetate (PMA)-stimulated leucocytes, ezrin is inactivated through calpain-mediated cleavage, moesin and radixin are insensitive to cleavage by calpain [41] suggesting that distinct regulatory mechanisms exist for each protein in the same cell.

### Interaction of ERMs with other proteins

There are several proteins within the plasma membrane that interact with activated ERM proteins through the FERM domain. In a manner dependent on PIP_2_, ERM proteins can associate with the cytoplasmic tails of intracellular adhesion molecules -1, -3 (ICAM-1 and -3) [42] and -2 (ICAM-2), as well as the hyaluronan receptor CD44 and CD43 [43]. ERMs are also known to bind PDZ (postsynaptic density protein)-containing proteins such as transporters and ion channels through other anchoring proteins like NHERF1 (Na^+^-H^+^ exchanger regulatory factor) also known as ERM-binding phosphoprotein 50 (EBP50) and NHERF2 [44, 45]. They also interact with membrane glycoproteins such as P-selectin glycoprotein ligand-1 which tether white blood cells to injured tissues [42]. The α-helical domain on the central portion of ERMs can also bind subunits of HOPS complex (homotypic fusion and protein sorting) as well as the regulatory subunits RII of protein kinase A [46]. Binding of ERM proteins to PKA tethers it to downstream targets to effect cAMP-mediated biological processes such as cell differentiation, proliferation, metabolism, apoptosis, exocytosis, T cell and B cell activation, muscle contraction [6]. In COS-1 cells, ezrin was shown to bind and link syndecan-2 to the cortical cytoskeleton [47]. The death receptor Fas/CD95, unlike other membrane proteins that bind all ERM proteins, did not bind to moesin but only to ezrin in T lymphocytes [48].

### ERM proteins and cancer

Cancer cell migration is a coordinated process involving different steps that bring about loss of cell-cell adhesion and deregulation of cell-matrix interaction. Several reports have outlined different factors such as localization of ERMs within the cell, their level of phosphorylation as well as expression profile to be responsible for ERM proteins-mediated promotion of tumourigenesis [2]. Below is a brief discussion of each of the ERM proteins, with the aim of highlighting their significance in different human cancers.

### Ezrin

Abnormal localization of ERM proteins is a leading factor resulting aberrant intracellular signal transduction triggered by growth factors. For instance, in breast carcinoma, ezrin which was originally situated at apical structures in normal cell was found translocated to the cytoplasm and plasma membrane and this aberrant localization resulted in the acquisition of an epithelial-mesenchymal transition (EMT) in which cells loss their normal differentiated, planar and apical-based polarity and anchorage dependent architecture and instead acquire metastatic phenotype that correlated with poor prognosis [49].

In epithelial cells, interaction of ezrin with Fes kinase causes recruitment and activation of the later at the cell membrane where it facilitates HGF-mediated loss of cell-cell and cell-ECM contacts resulting in cell migration as revealed by wound healing assay [32, 50]. In this interaction, ezrin not only localized to the leading edge of migrating epithelial cells [50], but also promoted the formation of membrane protrusions [2].

Similarly, upon phosphorylation of the ERM proteins by PKCα, ERMs can act as downstream effector of PKC to mediate cell migration when the later was stimulated with phorbol-ester [51]. PKC activation by phorbol ester also caused a switch in phosphorylation site of the transmembrane receptor CD44 from Ser325 to Ser29 and this phosphorylation regulated the association of ezrin with CD44 to promote directional cell migration triggered by CD44 [52]. Ezrin binds cell-neural adhesion molecule (L1-CAM) to promote progression of colorectal cancer in that RNA interference of ezrin activity inhibited tumour metastasis mediated by L1-CAM [53]. In 3D matrigel matrix, perturbation of ezrin activity with small hairpin RNA technology reduced the metastatic and invasive capabilities of MDA-MB-231 and MCF10A breast cancer cell lines [54]. Ezrin Y145 phosphorylation in mouse mammary carcinoma cell line SP1 [55] and in pig kidney epithelial LLC-PK1 [56] cells resulted in cell spreading. Elevated expression of ezrin has been reported in LTE, BE1, H446 and H460 lung cancer cell lines, and a substantial decrease in migration, proliferation and invasion was observed upon siRNA-mediated knockdown of ezrin [57]. In high grade prostate cancers, overexpression of ezrin has been reported [58, 59], and this was attributed to increased expression of oncogenic c-Myc [60]. Interestingly, ezrin itself, through a feedback loop involving the Akt/PI3K pathway can regulate c-Myc levels and this is crucial for cell migration and invasion [60, 61]. Ezrin overexpression has been shown in other cancer such as pancreatic carcinoma, rhabdomyosarcoma and osteosarcoma [62–64].

### Radixin

Although, much is not known about the role of radixin in cancer, unlike ezrin, however, radixin has been implicated in prostate cancer progression [65]; and impairment of radixin in human pancreatic cancer cell line by shRNA not only significantly attenuated cell proliferation, survival, adhesion and invasion but also enhanced expression levels of the cell-cell adhesion molecule, E-cadherin [66]. In a manner dependent on Vav (a guanine exchange nucleotide factor for Rac1) activity, downregulation of radixin levels resulted in an increase in Rac1 activity [67]. In radixin, phosphorylation of a conserved threonine 564 residue is sufficient to prevent the interaction of the FERM domain at the N-terminus with the F-actin binding domain at the C-ERMAD terminus, and this results in constitutive opening of the membrane and F-actin binding domains [68]. Indeed, in Madin-Darby canine kidney (MDCK) epithelial cells, phosphorylation of radixin on this site (T564) by the G protein-coupled receptor kinase 2 (GRK2) was able to induce membrane protrusions as well as increased migration of the cells as determined by wound healing assay [69]. In contrast to the aforementioned positive roles of radixin in tumourigenesis, a novel role in which the protein appeared to inhibit metastasis has been reported. According to this report, perturbation of radixin activity in the metastatic prostate cancer cell line PC3 by siRNA technology resulted in an elevated increase in spreading of cells, enhanced cell-cell adhesion and acquisition of epithelial phenotype [70].

### Moesin

Expression of moesin has been correlated with increased tumour size and invasive capability, and there was an aberrant trafficking of the protein from plasma membrane to the cytosol in oral squamous carcinoma cell (OSCC) in which moesin was knocked down [71]. Similarly, whereas high grade glioblastoma showed high expression levels of moesin, there was no change in expression levels of ezrin and radixin [72]. Moesin promoted tumour cell invasion in that in vitro 3D cell migration assays revealed that moesin depleted-cells exhibited reduced invasiveness [61, 73]. Moesin is considered an important promoter of metastasis as it has been shown to induce EMT in human mammary cell MCF10A [74], and there are now emerging reports that moesin is upregulated in different human cancer cell lines [75, 76] as well as a marker of EMT [74, 77, 78]. In the same vein, high level of moesin was also found in head and neck squamous cell carcinoma [79]. Whereas both moesin and radixin were found upregulated in lymph node metastases of pancreatic cancer, the level of ezrin expression was unaffected, but its phosphorylation status did change [80].

## Conclusion and future perspectives

ERM proteins play very vital role in maintaining cellular integrity and in mediating signal transduction from different extracellular inputs through their interaction different receptor tyrosine kinases (RTKs) such as EGFR and HGFR, adhesion and adaptor proteins such as E-cadherin, ICAM-1,2,3, NHERF and CD44, and other signaling pathways such as PI3K/Akt, cAMP/PKA and the Rho GTPases, all of which have been implicated in tumorigenesis; thus, making ERM proteins important target in development of novel therapeutics in fighting cancer progression. Although, there are ample reports on involvement of ERMs in cancer, detailed understanding of the mechanisms of their interactions with other proteins as well as their activation is still lacking and requires further investigation.

## Abbreviations

EGF: Epidermal growth factor
EMT: Epithelial-mesenchymal transition
ERM: Ezrin, radixin, moesin
FERM: Four point one ERM domain
HGF: Hepatocyte growth factor
ICAM: Intracellular adhesion molecule
LOK: Lymphocyte oriented kinase
NHERF: Na^+^-H^+^ exchanger regulatory factor
PIP_2_: Phosphatidylinositol 4,5-bisphosphate
PKA/C: Protein kinase A/C
RTKs: Receptor tyrosine kinases
S1P: Sphingosine-1-phosphate
